# Correction: 3D-printed electrochemical cells for multi-point aptamer-based drug measurements

**DOI:** 10.1039/d5sd90016f

**Published:** 2025-05-29

**Authors:** John Mack, Raygan Murray, Kenedi Lynch, Netzahualcóyotl Arroyo-Currás

**Affiliations:** a Biochemistry, Cellular and Molecular Biology Program, Johns Hopkins University School of Medicine 316 Hunterian Building, 725 North Wolfe Street Baltimore MD 21205 USA netzarroyo@jhmi.edu +1 443 287 4798; b Amgen Scholars Program, Krieger School of Arts and Sciences, Johns Hopkins University Baltimore MD 21218 USA; c Department of Pharmacology and Molecular Sciences, Johns Hopkins University School of Medicine Baltimore MD 21205 USA

## Abstract

Correction for ‘3D-printed electrochemical cells for multi-point aptamer-based drug measurements’ by John Mack *et al.*, *Sens. Diagn.*, 2024, **3**, 1533–1541, https://doi.org/10.1039/D4SD00192C.

We, the authors of the manuscript noted above, have identified an error in the text of the article. In Table 1, the DNA sequence for the tobramycin aptamer was mistakenly reported as 5′-GGC GAC AAG GAA AAT CCT TCA ACG AAG GTG GGT GGC C-3′.

The correct sequence for the tobramycin aptamer is given in the corrected Table 1 shown below, maintaining the original citation.

**Table 1:** DNA aptamer sequences

Name	Sequence
**Tobramycin** ^**13**^	**5′-GGG ACT TGG TTT AGG TAA TGA GTC CC-3′**
Vancomycin^1^	5′-CGA GGG TAC CGC AAT AGT ACT TAT TGT TCG CCT ATT GTG GGT CGG-3′
l-Procaine^14^	5′-GGC GAC AAG GAA AAT CCT TCA ACG AAG GTG GGT GGC C-3′
Irinotecan^11^	Proprietary (Aptamer Group, York, UK)

We apologize for this error, which occurred because of a copy-paste mistake during finalizing the manuscript draft. This mistake does not impact the significance, interpretation, or conclusion of the results of this study. However, we believe it important to clarify for the sake of transparency and repeatability.

The Royal Society of Chemistry apologises for these errors and any consequent inconvenience to authors and readers.

